# Impressions

**Published:** 1859-09

**Authors:** J. G. Hamill

**Affiliations:** Lancaster


### IMPRESSIONS.

MANY of the members of our profession, with a praiseworthy devotedness to the general good are contributing their experience in the art of taking good impressions of the mouth for the insertion of plates. Nearly all are agreed that plaster of paris and yellow wax are the best substances now in use for this purpose. Where all the teeth are out of the mouth, we use plaster, and think it very much superior to wax; but if some of the teeth are still standing, the difficulty experienced in the removal of it from the mouth drives us to the use of wax. The particular kind of cases to which we wish to call attention at this time, are those of very deep palatal arches, together with some of the natural teeth still standing.

We never could get so good impressions of such cases with anything else as wax. Our manner of procedure in such cases is as follows:

After having softened the wax, and placed it in an impression cup, after the usual manner of procedure for taking wax impressions, we take an additional lump of wax sufficiently large to fill the deepest part of arch—say that part bounded by the bicuspid and first molar teeth—and after making it quite soft, we wipe the roof of the mouth dry with a napkin, and press it up gently to its place. Then being careful not to allow it to get wet with the saliva, we hold that portion of the wax which is in the impression cup that will come in contact with that already in the mouth, over a spirit lamp until it is almost soft enough to run, then placing it in the mouth we press it up firmly against every part, which is to be covered by the plate. If the necessary precautions be taken, the two portions of the wax will unite, and can be removed all together, presenting a sufficiently good impression for a suction plate.

*Perhaps* somebody has taken impressions this way long ago, and have "*priority.*" If so, why didn't he tell us?

LANCASTER, July 18th, 1859. J. G. HAMILL.

